# Turpentine in Carbuncular Diseases

**Published:** 1855-11

**Authors:** 


Turpentine in Carbuncular Diseases.—Dr. Thielmann states that he has employed this substance with great success in a case of malignant pustule, and in a great number of cases of carbuncle, amounting to 342 since 1837. The treatment has been merely local, unless suppurative fever or other general symptoms called for interference. The turpentine was applied in every stage of the disease on a thick pad of charpie, evaporation being prevented by oiled silk. In most cases a slight burning is produced, which only lasts for a few minutes. The epidermis becomes softened, and the mortified parts are quickly separated, without the necessity of the crucial incision. After the seperation it is still continued, as under its influence the healing is rapid. If, after each dressing (these being repeated night and morning,) the patient complains of a continual burning, the lotion is to be sufficiently diluted with chamomile tea, or the dressing is to be performed with this alone. The turpentine is suitable to all sloughy and atonic ulcers. The following is the formula for the preparation of the application: Mix ℥j. of oil of turpentine with the yolk of an egg, and then add spirit of camphor ℥j., chamomile tea ℔j.—(*Berlin Medicin Zeitung.*) *London Lancet.*

